# Salicylic Acid Enhances Cadmium Tolerance in *Cornus alba* L. Seedlings Through Leaf Transcriptional Regulation and Enhanced Root Heavy Metal Sequestration

**DOI:** 10.3390/plants15071081

**Published:** 2026-04-01

**Authors:** Kai Qian, Te Li, Fan Huang, Tongbao Qu

**Affiliations:** College of Forestry and Grassland, Jilin Agricultural University, Changchun 130118, China; qiankai@jlau.edu.cn (K.Q.); 15140122381@163.com (T.L.); 18365105429@163.com (F.H.)

**Keywords:** Cd, SA, terpenoid biosynthesis, root immobilization

## Abstract

Salicylic acid enhances cadmium tolerance in plants by modulating antioxidant defenses and promoting cadmium immobilization in cell walls. However, its potential to mitigate cadmium-induced growth inhibition and physiological disturbances in the woody species *Cornus alba* L. remains unexplored. *Cornus alba* L. seedlings were used in the pot experiment with four treatments: control (CK); 40 mg·kg^−1^ cadmium treatment (Cd); 100 µmol·L^−1^ salicylic acid treatment (SA); and both salicylic acid and cadmium treatment (SACd). The results showed that salicylic acid reduced lipid peroxidation in cell membranes by enhancing root cadmium sequestration and reconfiguring the antioxidant enzyme system, thus demonstrating a synergistic protective effect. By inhibiting cadmium transport to the shoots, it thereby mitigated the cadmium-induced inhibition of photosynthesis and reproductive development. Transcriptome analysis indicated that salicylic acid upregulates key genes in sucrose and starch metabolism pathways (e.g., *TPS*, *GN1_2_3*, *otsB*), leading to enhanced carbon assimilation and energy supply. Furthermore, it upregulates the key terpenoid biosynthesis genes (including *HMGR* and *GGPS*), leading to a coordinated modulation of primary and secondary metabolic flux and an increased output of the related pathways. The results reveal a potential mechanism by which salicylic acid alleviates cadmium stress in *Cornus alba* L., offering new insights into its role in plant heavy metal stress responses.

## 1. Introduction

Rapid global industrialization and urbanization, coupled with inadequate emission controls, cause significant heavy metal contamination of soils [1,2]. Among these heavy metals, cadmium (Cd), a non-essential element for living organisms, is considered a primary pollutant owing to its high toxicity, environmental persistence, and strong bioaccumulation capacity, thus posing severe risks to both ecological balance and human health [3,4]. Cd stress adversely affects plant physiological processes primarily by disrupting the photosynthetic apparatus, damaging organelles, and leading to excessive accumulation of reactive oxygen species (ROS) [5]. These changes consequently trigger oxidative stress and competitively inhibit the uptake and translocation of essential nutrients in plants, including calcium (Ca), zinc (Zn), and iron (Fe) [6,7]. Such detrimental effects ultimately lead to a marked reduction in plant biomass and, in severe cases, even plant mortality, thereby threatening ecosystem stability. Therefore, developing efficient and sustainable strategies to mitigate Cd toxicity is a key focus in plant ecophysiology and soil remediation. Compared with physicochemical remediation approaches, the application of exogenous elicitors provides a more eco-friendly alternative. It is not only easy to implement but also can directly enhance plant tolerance to abiotic stress.

To mitigate Cd-induced toxicity in plants, current research has proposed various remediation strategies, mainly including physical remediation, chemical amendments, biological regulation, and genetic engineering approaches. Physical techniques such as soil washing efficiently remove contaminants directly, but their high cost and disruptive effects on soil structure restrict large-scale implementation. Chemical immobilization, often through amendments like lime or biochar, reduces Cd bioavailability, yet carries a potential risk of secondary pollution [8]. In contrast, exogenous regulators attract considerable attention due to their environmental friendliness, ease of application, and ability to directly enhance the plant stress resistance [9]. Previous studies demonstrate that foliar treatment with 24-epibrassinolide (BRs) improves Cd tolerance and phytoremediation efficiency in willow by upregulating genes associated with Ca channel activity, Zn/Ca transmembrane transport, antioxidant enzyme activity, glutathione metabolism, and vacuolar sequestration [10]. Under Cd stress, exogenous application of CaO nanoparticles (CaO NPs) significantly upregulates the expression of antioxidant enzyme-related genes in barley (*Hordeum vulgare* L.) seedlings, effectively enhancing plant biomass, antioxidant enzyme activities, and non-enzymatic antioxidant contents [11]. Abscisic acid (ABA) enhances Cd stress tolerance in pepper (*Capsicum annuum* L.) by modulating Cd distribution, activating antioxidant defense systems, and altering cell wall composition to reduce Cd uptake [12]. In Sea Barley (*Hordeum marinum* Huds.), exogenous silicon (Si) effectively alleviates Cd-induced growth inhibition and photosynthetic pigment degradation by suppressing Cd uptake, thereby reducing Cd accumulation in both roots and shoots [13]. Exogenous melatonin (MT) repairs Cd-induced DNA damage and upregulates the expression of genes involved in DNA repair pathways in tiger nut (*Cyperus esculentus* var. *sativus* Boeckeler) [14]. Additionally, exogenous iron (Fe) at appropriate concentrations promotes the formation of Fe plaques on the roots of *Avicennia marina* (Forssk.) Vierh., immobilizing Cd on the root surface and thereby reducing its uptake. This treatment also induces the secretion of low-molecular-weight organic acids (LMWOAs) from the roots, which enhance Cd detoxification through chelation or complexation [15]. Collectively, exogenous regulatory agents enhance plant tolerance and remediation capacity against Cd stress through multifaceted physiological, biochemical, and molecular mechanisms.

Salicylic acid [SA, formula: C_6_H_4_(OH)(COOH)] is a crucial phenolic growth regulator that plays a pivotal role in plant development [16,17]. It not only modulates core physiological processes including photosynthesis, ion uptake and translocation, but also mitigates diverse abiotic stresses [18]. SA has been widely documented to confer protection against heavy metal toxicity, including Zn stress in *Medicago sativa* L., Cd stress in *Triticum aestivum* L., and arsenic (As) stress in *Oryza sativa* L. [19,20,21]. Studies in *Brassica juncea* (L.) Czern. reveals that foliar application of SA not only improves growth parameters but also alleviates Cd-induced suppression of photosynthesis through regulation of stomatal aperture [22]. Importantly, SA also enhances the plant antioxidant defense mechanisms, thereby mitigating the toxic effects of Cd and other heavy metals. In Cd remediation studies using *Bacopa monnieri* (L.) Wettst., SA application not only significantly elevated the activities of superoxide dismutase (SOD), catalase (CAT), and peroxidase (POD), but also synergistically enhanced antioxidant capacity in combination with jasmonic acid (JA) [23]. Similarly, SA significantly enhances hexavalent chromium (Cr) tolerance in *Solanum lycopersicum* L. by increasing the activities of enzymes involved in the ascorbic acid-glutathione cycle and elevating non-enzymatic antioxidant concentrations under Cr stress [24]. Further evidence indicates that SA mediates plant responses to heavy metal stress mainly by modulating metal uptake, translocation, and tissue partitioning of metals, thereby alleviating their phytotoxic effects. For instance, in *Spinacia oleracea* L., SA limits Cd accumulation in shoots by downregulating key transporter gene expression, including *SoHMA4-like* and *SoNramp3.1-like* [25]. Studies on *Fagopyrum tataricum* (L.) Gaertn. confirm that SA plays an indispensable role in enhancing the root-mediated immobilization of heavy metals. SA treatment increased root Cd concentrations by 32% in *Fagopyrum tataricum* (L.) Gaertn., while drastically reducing shoot Cd levels. This restricted translocation, resulting from enhanced Cd retention in the roots, alleviated Cd-induced inhibition of photosynthesis and growth in the aboveground parts [26]. While SA is widely studied for mitigating Cd toxicity in herbaceous plants, its underlying mechanisms and practical applications in woody plants, particularly shrubs used in ecological restoration and landscaping, remain poorly understood. Woody plants differ substantially from herbaceous species in root system architecture, xylem transport dynamics, metal partitioning patterns and long-term adaptive strategies. Accordingly, clarifying the regulatory roles of SA in woody plant responses to Cd stress is critical for developing effective strategies to improve their stress resilience and growth performance in Cd-contaminated habitats.

*Cornus alba* L., a deciduous shrub species belonging to the genus Cornus within the family Cornaceae, is widely distributed across East Asia and Europe. It is characterized by vivid red stems that provide year-round ornamental appeal, establishing it as a valuable landscape shrub [27]. Existing studies on *Cornus alba* L. have mainly focused on genetic breeding and landscape utilization, while systematic research on its physiological responses and molecular regulatory mechanisms under stress remains limited [28,29]. Previous research has indicated that *Cornus alba* L. exhibits relatively high tolerance to drought and heavy metals compared with other shrub species [30,31,32]. Nonetheless, Cd stress severely inhibits its growth, causing leaf chlorosis and declined physiological function, which restricts its broader application in urban greening [27]. Therefore, identifying effective exogenous regulators to mitigate Cd toxicity in *Cornus alba* L. is crucial for enhancing its stress resistance and expanding its potential for ecological restoration. The primary objectives of this study are to (1) explore the physiological regulatory effects of foliar SA application on growth, Cd accumulation, photosynthetic capacity, and antioxidant defense systems in *Cornus alba* L. under Cd stress; and (2) analyze leaf transcriptomic changes to reveal the molecular mechanisms underlying SA-mediated improvements in heavy metal tolerance. In summary, the findings of this study aim to elucidate the potential mechanisms by which SA enhances Cd tolerance in *Cornus alba* L., providing crucial theoretical support for the rational application of SA in the ecological restoration of woody plants in Cd-polluted areas, the development of urban forestry, and the optimization of cultivation management strategies.

## 2. Results

### 2.1. Effects of SA Application on Growth Indices and Photosynthetic Parameters Under Cd Stress

**Figure 1:**
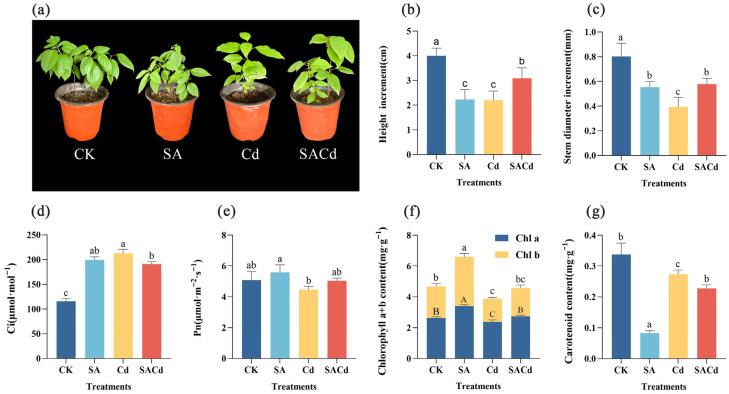
Effects of SA pretreatment on the growth indices, chlorophyll content and photosynthetic parameters of *Cornus alba* L. under Cd stress. (**a**) Morphology; (**b**) Height increment; (**c**) Stem diameter increment; (**d**) Intercellular CO_2_ concentration, (Ci); (**e**) Net photosynthetic rate, (Pn); (**f**) Concentrations of chlorophyll a and chlorophyll b, (Chl a + b); (**g**) Carotenoid content, (Caro). For height increment (**b**) and stem diameter increment (**c**): The values were measured before and after the treatment, and the growth increment was calculated as the difference between the post-treatment and pre-treatment measurements. Treatments: CK, control; Cd, cadmium; SA, salicylic acid pretreatment; SACd, salicylic acid pretreatment and cadmium. Different letters indicate significant differences between treatments (*p* < 0.05).

Exogenous SA significantly alleviated the inhibitory effect of Cd stress on the growth of *Cornus alba* L. Phenotypic observations showed a clear improvement in Cd-induced leaf chlorosis, and seedling growth generally exhibited a positive trend in the SACd compared to the Cd (Figure 1a). Compared to the CK, Cd stress reduced plant height increase by 45% and stem diameter increase by 51%. In contrast, the SA application markedly alleviated these negative effects, leading to significant improvements in both height and stem diameter growth in the SACd (Figure 1b,c).

Cd stress significantly suppressed photosynthetic function, as shown by declining photosynthetic parameters and altering pigment composition (Figure 1d–g). The result showed that the net photosynthetic rate (Pn) decreased, and the contents of Chlorophyll a (Chl a), Chlorophyll b (Chl b), and Carotenoid (Caro) were significantly lower than those of the CK (*p* < 0.05, Table S2). Concurrently, impaired carbon assimilation led to the accumulation of intercellular CO_2_ concentration (Ci). Following SA application, Ci decreased significantly compared to Cd, while Pn displayed a pronounced recovery (*p* < 0.05, Table S2). The contents of Chl a and Chl b not only significantly exceeded in the Cd but also recovered to levels comparable to the CK. However, the Caro content decreased further.

### 2.2. Effects of SA Application on Antioxidant-Related Parameters and Osmoregulatory Substances Under Cd Stress

**Figure 2:**
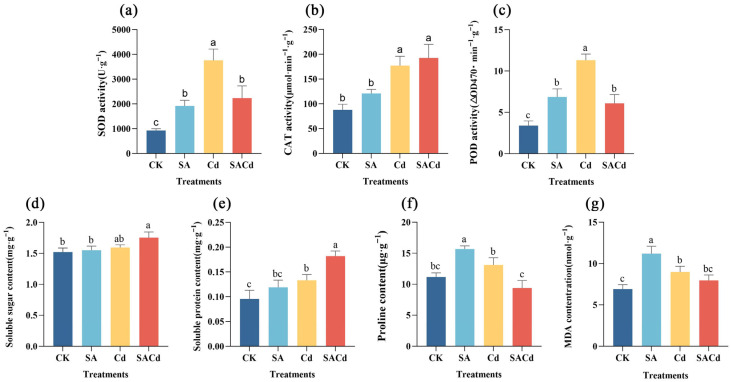
Effects of SA pretreatment on physiological indices of *Cornus alba* L. under Cd stress. (**a**) Superoxide dismutase (SOD) activity, (**b**) Catalase (CAT) activity, (**c**) Peroxidase (POD) activity, (**d**) Soluble sugar (SS) content, (**e**) Soluble protein (SP) content, (**f**) Proline (Pro) content, (**g**) Malondialdehyde (MDA) content. Treatments: CK, control; Cd, cadmium; SA, salicylic acid pretreatment; SACd, salicylic acid pretreatment and cadmium. Different lowercase letters indicate significant differences between treatments (*p* < 0.05).

Compared to the CK, the exposure to Cd stress significantly enhanced the activities of SOD, CAT, and POD, along with the malondialdehyde (MDA) content in leaves (*p* < 0.05, Figure 2a–c,g, Table S3). Additionally, the levels of osmotic regulatory substances, namely proline (Pro), soluble sugar (SS), and soluble protein (SP), were also substantially elevated under Cd stress (*p* < 0.05, Figure 2d–f, Table S3).

Under non-stress conditions, exogenous SA significantly increased the levels of lipid peroxidation-related markers and osmotic substances (*p* < 0.05, Table S3). Notably, compared to the Cd, the SA application resulted in a significant decline in SOD activities, POD activities, and MDA content, whereas CAT activity was uniquely up-regulated (*p* < 0.05, Figure 2a–c,g, Table S3). Moreover, the accumulation of SS and SP was further enhanced under SACd, with both parameters being significantly higher than those in the CK. The accumulation trend of Pro was diametrically opposed to that of SS and SP (Figure 2d–f).

### 2.3. Effects of SA Application on Cd Accumulation, Bioconcentration Factor and Transport Coefficient Under Cd Stress

Heavy metal analysis revealed no significant difference in Cd accumulation between the CK and SA in *Cornus alba* L. (Table 1). Cd stress led to significant Cd accumulation in the root system of *Cornus alba* L. Under the SACd, Cd content in the shoots decreased significantly by 28.1% compared to the Cd, while Cd content in the roots increased significantly by 99.1% (*p* < 0.05, Table S4).

Bioconcentration Factor (BCF) and Translocation Factor (TF) are the key indicators for assessing the capacity to absorb, accumulate, and transport heavy metals of plants. Compared to the CK, the BCF value was significantly elevated in both the Cd (1.5-fold higher) and SACd (3.8-fold higher) treatments (*p* < 0.05, Table S4). Regarding the TF, a slight increase was observed in the SA treatment compared to CK. Conversely, TF decreased markedly in the Cd and SACd treatments, reaching values of 0.0021 and 0.0008, respectively, both significantly lower than CK (*p* < 0.05, Table S4). No significant difference in TF was observed between the Cd and SACd.

### 2.4. SA Modulates Transcriptomic Responses in Cornus alba L. Leaves Under Cd Stress

#### 2.4.1. Transcriptome Sequencing Data and Quality Assessment

**Figure 3:**
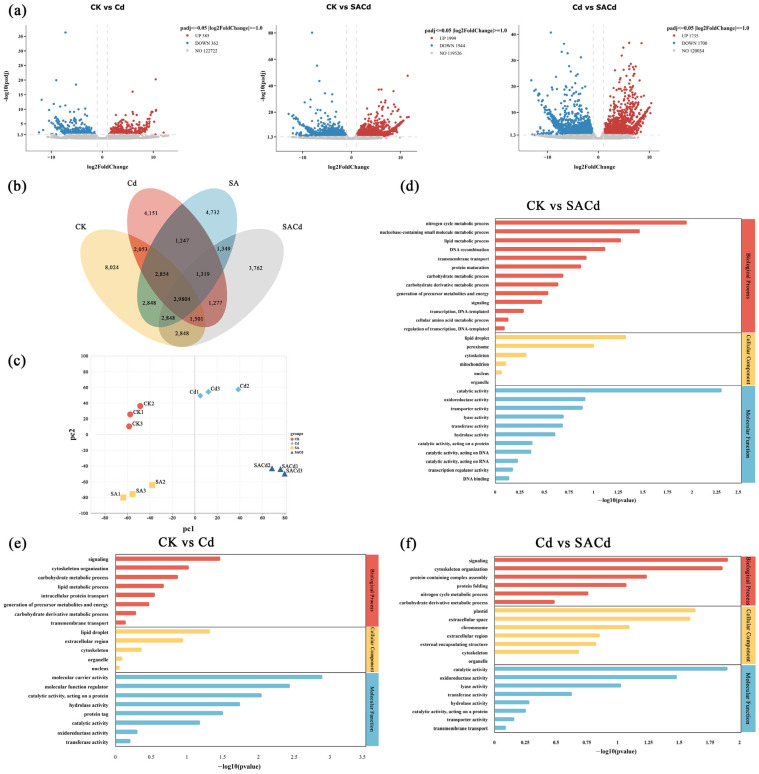
DEGs in the leaves of *Cornus alba* L. under Cd stress with SA pretreatment. (**a**) Volcano plot illustrating DEGs in the leaves of *Cornus alba* L. The blue dots indicate downregulated genes, the red dots indicate upregulated genes, and the gray dots indicate genes whose expression did not significantly change. (**b**) Plot of principal component analysis of transcriptome data of the treatments of CK, SA, Cd, and SACd. (**c**) Venn diagram of DEGs under different treatments. (**d**) GO enrichment analysis of the DEGs coexpressed in CK and SACd. (**e**) GO enrichment analysis of the DEGs coexpressed in CK and Cd. (**f**) GO enrichment analysis of the DEGs coexpressed in Cd and SACd.

Transcriptome sequencing was conducted on *Cornus alba* L. leaf samples, with 12 data libraries constructed for RNA-Seq analysis, generating approximately 30.60 GB of raw data. To ensure data quality for downstream analysis, raw reads were processed to obtain high-quality clean reads. The number of clean reads per library ranged from 55.91 million to 63.39 million. Principal component analysis (PCA) revealed robust clustering of biological replicates and a well-defined separation between experimental groups, confirming the high quality and reliability of the dataset for subsequent transcriptomic analysis (Figure 3c). Furthermore, all samples exhibited high sequencing quality, with GC content above 43%, Q20 values greater than 99%, and Q30 values exceeding 97% (Table S1). These indices verified that the transcriptomic sequencing data were of high quality and suitable for subsequent differential expression and enrichment analyses.

#### 2.4.2. Analysis of Differentially Expressed Gene Numbers

**Figure 4:**
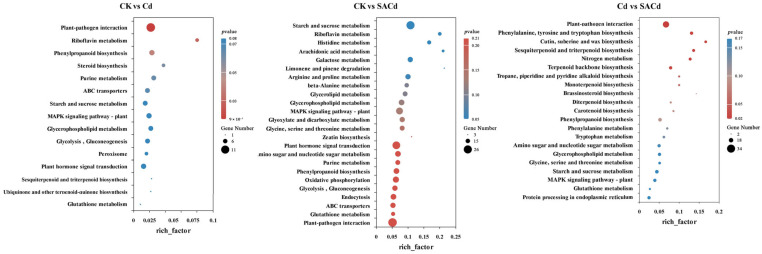
KEGG analysis of the DEGs coexpressed in the leaves of *Cornus alba* L. under CK, Cd, and SACd.

Venn diagram analysis identified 29,804 genes that were commonly differentially expressed in (Figure 3b). A significantly higher number of Differentially Expressed Genes (DEGs) was identified not only in the CK vs. SACd and but also in Cd vs. SACd comparisons relative to all the other pairwise comparisons in *Cornus alba* L. Pairwise comparisons between different groups revealed distinct gene expression patterns. Specifically, the CK vs. Cd comparison identified 539 DEGs, comprising 288 upregulated and 251 downregulated genes. The CK vs. SACd comparison yielded 3943 DEGs (1999 upregulated and 1944 downregulated), while the Cd vs. SACd comparison detected 3851 DEGs (1984 upregulated and 1867 downregulated) (Figure 4).

#### 2.4.3. GO and KEGG Enrichment Analysis

Gene Ontology (GO) enrichment analysis revealed significant functional categories (*p* < 0.05) that were enriched with DEGs in each comparison (Figure 3d–f). In the CK vs. Cd comparison, DEGs were enriched in 95 GO terms, comprising 47 biological processes (BP), 22 cellular components (CC), and 26 molecular functions (MF). Key enriched terms included ‘signaling’ (GO: 0023052), ‘extracellular region’ (GO: 0005576), ‘molecular function regulator’ (GO: 0098772), and ‘catalytic activity, acting on a protein’ (GO: 0140096). For the Cd vs. SACd comparison, 118 GO terms (60 BP, 27 CC, 31 MF) were significantly enriched, including ‘signaling’ (GO: 0023052), ‘cytoskeleton organization’ (GO: 0007010), ‘catalytic activity’ (GO: 0003824), and ‘oxidoreductase activity’ (GO: 0016491). The CK vs. SACd comparison showed the highest number of enriched 122 GO terms (61 BP, 28 CC, 33 MF), with prominent categories such as ‘catalytic activity’ (GO: 0003824), ‘nucleobase-containing small molecule metabolic process’ (GO: 0055086), ‘lipid metabolic process’ (GO: 0006629), ‘transmembrane transport’ (GO: 0055085), and ‘oxidoreductase activity’ (GO: 0016491).

Kyoto Encyclopedia of Genes and Genomes (KEGG) pathway identified significantly enriched pathways associated with the treatments (Figure 4). Cd stress significantly affected multiple pathways, including ‘Plant-pathogen interaction’ (ko04626), ‘Phenylpropanoid biosynthesis’ (ko00940), ‘MAPK signaling pathway-plant’ (ko04016), ‘ABC transporters’ (ko02010), ‘Starch and sucrose metabolism’ (ko00500), and ‘Plant hormone signal transduction’ (ko04075). In contrast, exogenous SA application primarily influenced pathways related to ‘Plant-pathogen interaction’ (ko04626), ‘Phenylalanine, tyrosine and tryptophan biosynthesis’ (ko00400), ‘Sesquiterpenoid and triterpenoid biosynthesis’ (ko00909), ‘Terpenoid backbone biosynthesis’ (ko00900), ‘Starch and sucrose metabolism’ (ko00500), and ‘MAPK signaling pathway-plant’ (ko04016).

### 2.5. SA Modulates Gene Transcription Levels in Secondary Metabolic Pathways

#### 2.5.1. SA Modulates Biological Pathways Associated with Terpenoid Biosynthesis

**Figure 5:**
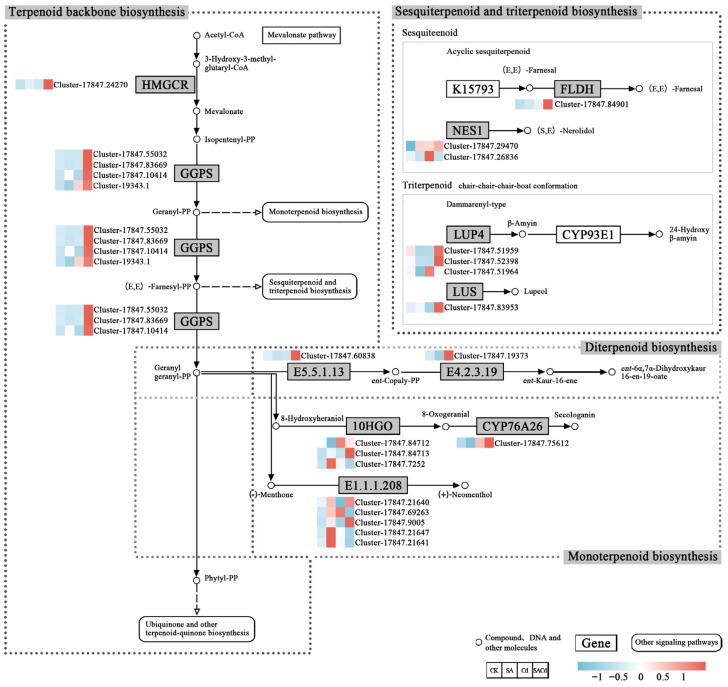
Analysis of pathway map of Sesquiterpenoid and triterpenoid biosynthesis, Terpenoid backbone biosynthesis, Monoterpenoid biosynthesis and Diterpenoid biosynthesis. The red block represents up-regulated genes, the blue block represents down-regulated genes, and the white block represents all genes’ average.

Cd stress significantly suppressed the expression of the genes associated with terpenoid biosynthesis pathways (Figure 5), whereas SA application significantly upregulated the expression of multiple key genes (*p* < 0.05). In the terpenoid backbone biosynthesis pathway, SA treatment significantly upregulated the key genes, including *HMGCR* and *GGPS*, which encode enzymes critical for terpenoid precursor synthesis.

SA also significantly enhanced the expression of genes in downstream monoterpene and diterpene biosynthesis pathways, including the genes encoding ent-copalyl diphosphate synthase (E5.5.1.13), ent-kaurene synthase (E4.2.3.19), (+)-neomenthol dehydrogenase (E1.1.1.208), and 10-hydroxygeraniol oxidoreductase (10HGO), thereby promoting the synthesis of terpenoid derivatives. Similarly, in the sesquiterpene and triterpene biosynthesis pathways, the expression levels of *FLDH*, *NES1*, *LUP4*, and *LUS* were significantly higher under SA treatment than under Cd stress. These genes encode enzymes involved in the production of stress-protective terpenoids, including nerolidol and lupeol, which play key roles in plant defense responses and the maintenance of membrane stability.

#### 2.5.2. SA Modulates Starch/Sucrose Metabolism

**Figure 6:**
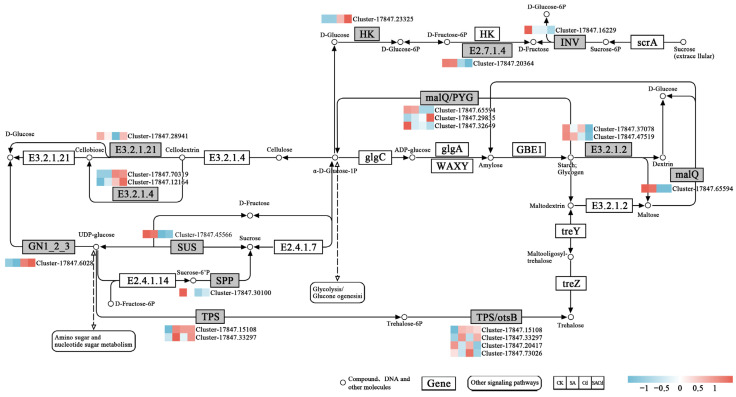
Analysis of the pathway map of Starch and sucrose metabolism. The red block represents up-regulated genes, the blue block represents down-regulated genes, and the white block represents all genes’ average.

Cd stress led to significant repression of multiple genes associated with starch and sucrose metabolism. Conversely, the SA application resulted in the upregulation of several genes within this pathway, such as *HK*, *bgIB*, *GN1_2_3*, *TPS*, *otsB*, *malQ*, and *PYG* (Figure 6). These genes are involved in sugar sensing, trehalose biosynthesis, and starch degradation, contributing to energy homeostasis and osmotic adjustment under stress conditions.

### 2.6. RT-qPCR Validation of Transcriptome Data

To validate the transcriptomic profiling data, the expression levels of four selected DEGs were quantified using quantitative real-time polymerase chain reaction (RT-qPCR). Compared with Cd stress alone, exogenous SA treatment significantly upregulated the expression of these four key genes which are involved in distinct metabolic pathways. These genes participate in terpenoid backbone biosynthesis (*Cluster*-17847.24270), triterpenoid defensive metabolism (*Cluster*-17847.83953), monoterpenoid derivative production (*Cluster*-17847.9005), and glycometabolic regulation (*Cluster*-17847.28941), collectively validating the molecular network underlying SA-mediated Cd stress mitigation from multiple perspectives. The expression trends from RNA-seq were consistent with those detected by qPCR (Figure S1), confirming that the transcriptomic data obtained in this study are highly reliable and accurate.

### 2.7. Correlation Analysis of Physiological Indicators and Related Genes Under Different Treatments

**Figure 7:**
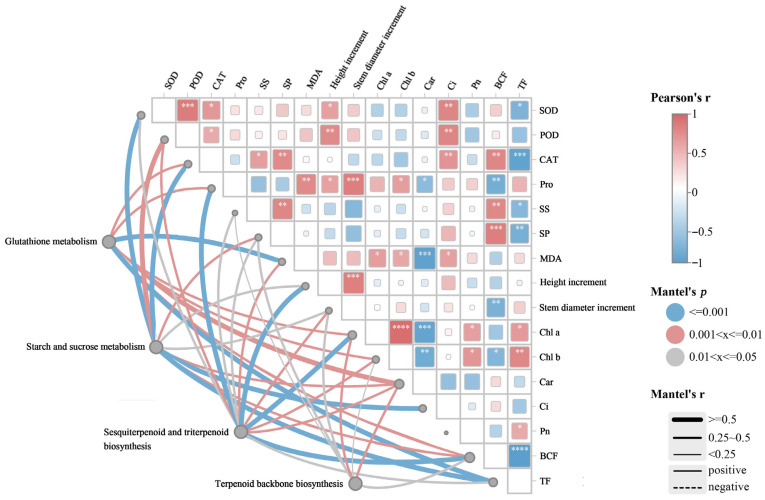
Mantel test correlation study between physicochemical indices and related genes in the growth of *Cornus alba* L. The lines represent the correlation between related genes and physicochemical indices, with thicker lines indicating stronger correlations. Solid lines: positive correlations. Dotted lines: negative correlations. *: *p* < 0.05, **: *p* < 0.01, and ***: *p* < 0.001, ****: *p* < 0.0001.

Pearson correlation analysis revealed significant relationships between growth and physiological indicators in *Cornus alba* L. (Figure 7). The height increment was positively correlated with antioxidant enzyme activities (SOD, POD) and Pro content (*p* < 0.05). The BCF exhibited a significant positive correlation with osmotic regulators (SP, SS) and CAT activity (*p* < 0.05), whereas it was negatively correlated with photosynthetic pigment content (Chl a and Chlb) (*p* < 0.05).

Mantel test analysis revealed significant correlations between transcribed genes and physicochemical indicators in *Cornus alba* L. (Figure 7). DEGs involved in glutathione metabolism were significantly correlated with MDA content and TF (r < 0.6, *p* = 0.001), as well as with CAT activity, Pro content, BCF, and photosynthetic pigment content (r < 0.6, *p* < 0.007). DEGs related to starch and sucrose metabolism were strongly correlated with antioxidant enzyme activity and Cd enrichment transport coefficients (r < 0.7, *p* < 0.003), and were also linked to increases in plant height and stem diameter (r < 0.4, *p* < 0.03). Furthermore, DEGs in the terpenoid biosynthesis pathway were correlated with both photosynthetic pigment content and BCF (r < 0.6, *p* < 0.05), while the genes in sesquiterpene and triterpene biosynthesis pathways were associated with plant height, stem diameter increment, POD activity, and Pro content (r < 0.6, *p* < 0.005). Collectively, the integrated analysis reveals that SA-induced enhancement of Cd tolerance in *Cornus alba* L. is associated with concurrent improvements in the antioxidant system, photosynthetic capacity, and a reduction in Cd accumulation. These physiological responses are strongly correlated with the differential expression of genes involved in plant secondary metabolism.

## 3. Discussion

SA alleviates Cd-induced growth inhibition, photosynthetic impairment, and chlorophyll degradation, and furthermore, improves plant tolerance to abiotic stress [33,34]. In this study, Cd stress caused a significant decrease in photosynthetic pigment (Chla, Chlb, and Caro) content in Cornus alba L. leaves (Figure 1), resulting in reduced light energy capture efficiency and causing leaf chlorosis directly. These studies are consistent with some previous research findings [2,35,36]. The concurrent sharp decline in Pn and abnormal increase in Ci indicate that Cd stress primarily impedes photosynthesis through non-stomatal limitations, potentially involving factors such as chloroplast structural damage, impaired photosystem function, or disrupted photosynthetic electron transport chains [37,38]. Following SA treatment, the contents of Chl a, Chl b, and Pn were all significantly recovered (Figure 1), indicating that SA mitigates Cd stress damage to the photosynthetic apparatus at the levels of both chlorophyll synthesis and photosynthetic activity. This preserves the plant’s carbon assimilation capacity, thus facilitating growth recovery [33]. These results directly account for the alleviation of leaf chlorosis and the marked improvement in overall plant growth observed under SA treatment. Notably, SA treatment accelerated carotenoid degradation, leading to further reductions in its content. This effect aligns with prior observations in *Linum usitatissimum* L. and *Ziziphus jujuba* var. *Spinosa* (Bunge) Hu ex H.F.Chow [39,40]. The reduction in carotenoid content observed after SA application under Cd stress may be explained by two potential mechanisms. First, SA likely restores total chlorophyll levels, thereby enhancing light energy utilization and reducing the need for carotenoids for non-photochemical quenching. Second, SA may activate complementary antioxidant defense systems, which alleviate oxidative pressure on carotenoids and decrease their demand for reactive oxygen species scavenging.

Cd absorption and translocation vary substantially among plant species and tissues, with roots serving as the main barrier against soil Cd and a key regulator of its subsequent distribution within the plant [41,42,43]. In the present study, Cd accumulation was significantly higher in the roots of *Cornus alba* L. than in its shoots (Table 1), indicating that the roots possess a strong capacity for Cd retention and sequestration. Consistent with the previous studies, the characteristic has been observed in *Symphytum officinale* L. and *Morus alba* L. [44,45]. Exogenous application of SA further enhanced the Cd retention capacity in *Morus alba* L. This reinforcement is likely attributable to SA-induced Cd sequestration in root cell walls and vacuoles, which limits its translocation to aboveground tissues, thereby reducing the potential for Cd to interfere with key metabolic processes [46]. Such containment of Cd within the roots allows *Morus alba* L. to sustain photosynthesis and biomass production under Cd stress, reinforcing internal detoxification while simultaneously limiting Cd accumulation in shoots and grains [46,47]. Additionally, the Cd concentration in the aboveground tissues of *Cornus alba* L. was significantly lower than that in the soil, with TF well below 1, indicating its characteristic as a metal excluder plant. This result can be primarily attributed to the restricted translocation of heavy metal ions from roots to shoots [16,48]. Moreover, SA treatment may activate a long-distance signaling pathway, which transmits signals perceived by the leaves to the root system, ultimately enhancing Cd compartmentation in *Cornus alba* L. [49,50].

In this study, the significant accumulation of Pro under Cd stress (Figure 2) represents a typical plant stress response to heavy metal toxicity, aimed at mitigating cellular damage through osmotic regulation and ROS scavenging [51,52]. However, exogenous application of SA reversed this trend, leading to a marked reduction in Pro content compared to Cd treatment alone (Figure 2). This result is in line with previous reports in pea (*Pisum sativum* L.) [34] and soybean [*Glycine max* (L.) Merr.] [53], contrast with findings reported in sunflower (*Helianthus annuus* L.) [54], banana (*Musa acuminata* Colla) [55] and other plant species. This may be attributed to SA-enhanced Cd retention and sequestration in the roots, which fundamentally alleviates osmotic stress and oxidative damage in plant cells, thereby reducing the need to maintain high proline levels at the cost of glutamate and nicotinamide adenine dinucleotide phosphate (NADPH) [51]. Transcriptomic analysis revealed that SA treatment activated the starch and sucrose metabolism pathway in the leaves (Figure 6), accompanied by a significant upregulation of the genes encoding trehalose-phosphate synthase (*TPS*), trehalose-phosphatase (*otsB*), glucan 1,3-β-glucosidase (*GN1_2_3*), and β-glucosidase (*Cluster*-17847.28941). The synergy between *TPS* and *otsB* enhances trehalose synthesis, concomitant with the upregulation of *Cluster*-17847.28941, which accelerates starch degradation [56]. This coordinated regulatory network ultimately drives a significant accumulation of carbohydrate, particularly soluble sugars, in the leaves. The accumulation of soluble sugars not only serves as a more stable osmolyte to maintain turgor pressure but, more importantly, supplies essential energy (ATP) and carbon skeletons for SA-induced defense responses and damage repair [57,58]. Similarly, the increase in soluble protein content likely reflects enhanced synthesis of functional proteins associated with stress adaptation and metabolic recovery. Together, the elevated levels of sugars and functional proteins provide both material resources and (in the case of sugars) signaling support, facilitating more effective cadmium sequestration in roots. This systemic process ultimately alleviates cadmium toxicity in leaves and reproductive organs.

Despite the consistent role of exogenous SA in ameliorating Cd oxidative stress via redox balancing, the underlying regulatory pathways are highly complex and species-dependent [59]. We found that Cd stress significantly elevated MDA content in *Cornus alba* L. leaves (Figure 2), suggesting that excessive ROS accumulation triggered membrane lipid peroxidation and caused severe damage to the cell membrane system [18]. As a stress response, *Cornus alba* L. activates antioxidant enzymes such as SOD, POD, and CAT to mitigate excess ROS and maintain intracellular redox balance, as reported in plants like *Pterocarya fraxinifolia* (Poir.) Spach and *Sedum alfredii* Hance [18,60]. Unlike typical antioxidant responses, SA treatment in *Cornus alba* L. was not associated with an enhancement of SOD and POD activity; instead, it significantly reduced the activity of both enzymes (Figure 2). This suggests that SA alleviates Cd-induced oxidative stress not through the conventional antioxidant enzyme system, but rather via a unique regulatory mechanism. Correspondingly, the content of MDA decreased, indicating that the oxidative stress state induced by Cd stress had been effectively alleviated [61]. Transcriptome data analysis suggests this phenomenon may stem from SA’s profound regulation of the terpenoid biosynthesis pathway. The expression levels of genes encoding key enzymes in the terpenoid skeleton biosynthesis pathway, including *HMGR* and *GGPS*, were substantially upregulated (Figure 5). This increase consequently enhanced the production of phytyl pyrophosphate (phytyl-PP). As a key precursor for vitamin E and chlorophyll synthesis, phytyl-PP alleviates Cd-induced oxidative damage by contributing to the formation of the antioxidant system and stabilizing photosynthetic structures [62,63]. Concurrently, the expression of key enzyme-encoding genes in the downstream pathways for monoterpene, sesquiterpene, diterpene, and triterpene biosynthesis was also significantly upregulated (Figure 5). This coordinated upregulation suggests a comprehensive activation of the overall terpenoid biosynthetic pathway. As critical natural antioxidants, terpenoids synthesized by activated biosynthetic pathways exert a direct ROS-scavenging effect through non-enzymatic mechanisms; when coupled with the enzymatic antioxidant system, this non-enzymatic action elicits a synergistic enhancement of cellular tolerance to oxidative stress [64]. Notably, CAT activity exhibited distinct changes compared to SOD and POD, showing further elevation following SA treatment (Figure 2). This discrepancy may relate to excessive H_2_O_2_ accumulation within plants [65]. Given that CAT is the primary enzyme responsible for H_2_O_2_ mitigation in plants, while SA-induced terpenoids preferentially target superoxide anions, the detoxification of H_2_O_2_ detoxification falls primarily on CAT, leading to its sustained upregulation [66]. This functional reallocation within the antioxidant enzyme system, coupled with synergistic interactions between enzymatic and non-enzymatic antioxidant pathways, underlies the core mechanism by which SA enhances Cd tolerance in *Cornus alba* L.

## 4. Materials and Methods

### 4.1. Plant Material and Growth Conditions

One-year-old *Cornus alba* L. seedlings were provided by the Senyuan Nursery in Kaiyuan City, and the experiment was conducted in the Jilin Agricultural University Artificial Climate Chamber (125°24′58″ E, 43°48′37″ N). The seedlings were acclimatized in a sterilized growth substrate within an artificial greenhouse at the experimental base. Daytime temperature was maintained at 26 ± 1 °C and nighttime temperature at 20 ± 1 °C. A photoperiod of 8 h light (8000 lx) and 16 h dark was applied daily. Relative humidity was kept between 50% and 70%.

### 4.2. Experimental Designs

The concentration of SA for foliar spray was selected as 100 μmol·L^−1^ according to preliminary experiments, which showed its optimal efficacy in promoting the growth of *Cornus alba* L. under Cd stress at 40 mg·kg^−1^. All reagents used were of analytical grade. Specifically, salicylic acid (C_7_H_6_O_3_) and cadmium chloride hemipentahydrate (CdCl_2_·2.5H_2_O) were purchased from Changchun Anmei Biotechnology Co. (Changchun, China). After the seedlings had developed 3–4 true leaves, individuals with uniform growth were selected and randomly assigned to four groups. Seedlings were then transplanted into wide-mouth pots (17.5 cm in height × 20.5 cm in diameter). Each pot was planted with three seedlings, and three biological replicates were established per treatment group. Each pot was planted with three seedlings as one biological replicate, and three biological replicates (i.e., three pots) were set up per treatment group, giving a total of 12 pots. The specific processing group included control (CK), 40 mg·kg^−1^ cadmium treatment (Cd), 100 µmol·L^−1^ salicylic acid treatment (SA), and both salicylic acid and cadmium treatment (SACd). Prior to the experimental setup, the soil was treated for one month with distilled water or a Cd solution at 40 mg kg^−1^ to achieve Cd fixation. Plants in the SA and SACd were treated with foliar sprays of 100 mL of a 100 μmol·L^−1^ SA solution per pot, ensuring all three seedlings in the pot were thoroughly sprayed. Plants were sprayed once every two days for a total of five applications. Morphological and relevant physiological indices were determined 30 days after the last SA application. Concurrently, a subset of plant samples was collected, flash-frozen in liquid nitrogen, and stored at −80 °C for later transcriptome sequencing analysis.

### 4.3. Indicator Measurement

#### 4.3.1. Measurement of Growth Indices

Thirty days after the final SA application, *Cornus alba* L. plants were harvested and separated into aboveground and underground portions at the root collar. The height of the aboveground part was measured using a tape measure. Stem diameter was determined at a position 2 mm above the soil surface using a vernier caliper. Plant height and stem diameter measurements were taken both before and after SA spraying. The differences between the pre- and post-treatment measurements were calculated and used as indices of growth increment.

#### 4.3.2. Measurement of Photosynthetic Physiological Indices

Pn and Ci were determined using a portable photosynthesis system (Li-6400, Li-Cor, Lincoln, NE, USA). CO_2_ concentration was controlled via a closed air circuit equipped with an internal light source and a cylindrical chamber. The CO_2_ concentration was set at 400 μmol·mol^−1^, the flow rate at 500 μmol·s^−1^, and the photosynthetic photon flux density (PPFD) at 1200 μmol·m^−2^·s^−1^. The ambient temperature was maintained at 25 °C, and the relative humidity at 70%. Measurements were conducted on three plants per treatment on sunny mornings [67].

Chlorophyll content was quantified using an ethanol–acetone mixture extraction method. Fresh leaf samples were immersed in 10 mL of a 1:1 (*v*/*v*) mixture of 95% ethanol and 80% acetone, with incubation in darkness during extraction. The absorbance of the pigment extract was determined at 665 nm, 649 nm, and 470 nm using a spectrophotometer to calculate photosynthetic pigment content [68].

#### 4.3.3. Measurement of Antioxidant Enzyme Activities

The activities of POD and CAT were determined using commercial assay kits purchased from Suzhou Grace Biotechnology Co., Ltd. (Suzhou, China). Fresh leaf samples (0.1 g) were ground into powder in liquid nitrogen and homogenized with 1 mL of extraction buffer in an ice bath. The homogenate was centrifuged at 12,000 rpm for 10 min at 4 °C, and the supernatant was collected and kept on ice for subsequent measurement. The reaction mixture was prepared following the manufacturer’s instructions. The absorbance was measured at 470 nm (for POD) and 240 nm (for CAT) using a UV-Vis spectrophotometer, respectively. Enzyme activities were calculated based on the standard curve and calculation formula provided in the kit.

SOD activity was determined using a commercial kit obtained from Beijing Solarbio Science & Technology Co., Ltd. (Beijing, China). Fresh leaf samples (0.1 g) were ground into powder in liquid nitrogen and homogenized with 1 mL of extraction buffer in an ice bath. After centrifugation at 8000 rpm for 10 min at 4 °C, the supernatant was collected and stored on ice until analysis. The reaction system was prepared according to the kit protocol, mixed thoroughly, and incubated in a water bath at 37 °C for 30 min. The absorbance was detected at 450 nm using a UV-Vis spectrophotometer, and SOD activity was calculated using the standard curve and calculation formula supplied with the kit.

#### 4.3.4. Measurement of Osmoregulatory Substances and Malondialdehyde Content

The SS content was determined using the anthrone colorimetric method [68]. Fresh leaf samples (0.1 g) were ground into powder in liquid nitrogen, and 0.8 mL of 80% (*v*/*v*) ethanol was added. The mixture was homogenized thoroughly under ice-bath conditions. The homogenate was transferred to an Eppendorf tube, and the volume was adjusted to 1.5 mL with 80% (*v*/*v*) ethanol. The homogenate was incubated in a water bath at 50 °C for 20 min, cooled to room temperature, and then centrifuged at 12,000 rpm for 10 min. A 25 μL aliquot of the supernatant was collected, followed by the addition of 75 μL of distilled water and 30 μL of anthrone reagent. Then, 250 μL of concentrated sulfuric acid was slowly added, and the mixture was mixed thoroughly. The reaction mixture was heated in a water bath at 95 °C for 10 min, cooled to room temperature, and the absorbance was measured at 620 nm. The soluble sugar content was calculated accordingly.

The SP content was determined by the Coomassie Brilliant Blue G-250 staining method [69]. Fresh leaf samples (0.1 g) were ground into a fine powder in liquid nitrogen, homogenized with 1 mL of phosphate buffer, and then centrifuged at 12,000 rpm for 10 min at 4 °C. An aliquot of 160 μL of the supernatant was mixed with 800 μL of Coomassie Brilliant Blue staining solution, and colorimetric measurement was performed at a wavelength of 600 nm to calculate the soluble protein content.

The Pro content was determined using the ketone-indole colorimetric method [68]. Fresh leaves (0.1 g) were ground into powder with liquid nitrogen and homogenized thoroughly with 1 mL of ice-cold 3% sulfosalicylic acid. The homogenate was heated at 90 °C for 10 min with shaking, and then centrifuged at 12,000 rpm for 10 min at room temperature. The supernatant was collected and cooled. Then, 600 μL of the cooled supernatant was mixed thoroughly with 300 μL of distilled water, 300 μL of glacial acetic acid, and 300 μL of 3% sulfosalicylic acid. The mixture was heated at 95 °C for 30 min. After cooling, the absorbance at 520 nm was measured, and the proline content was calculated based on the standard curve.

The MDA content was determined using the thiobarbituric acid reaction (TBARS) method [70]. Fresh leaves (0.1 g) were ground into powder with liquid nitrogen and homogenized thoroughly with 1 mL of 5% trichloroacetic acid (TCA). The homogenate was then centrifuged at 12,000 rpm for 15 min at 4 °C. Subsequently, 200 μL of the supernatant was mixed with 300 μL of 0.5% thiobarbituric acid (TBA) solution. The mixture was heated in a water bath at 95 °C for 30 min, followed by rapid cooling on ice, and then centrifuged at 12,000 rpm for 10 min. The absorbance of the supernatant was measured at 532 nm and 600 nm, and MDA content was calculated accordingly.

All of the reagents mentioned above were purchased from Changchun Anmei Biotechnology Co. (Changchun, China).

#### 4.3.5. Measurement of Cadmium Content

Thirty days after the final foliar application of SA, plant samples of *Cornus alba* L. were collected. The aboveground parts (leaves and stems) and belowground parts (roots) were collected separately, and rhizosphere soil was simultaneously gathered and sieved. Fresh samples were immediately snap-frozen in liquid nitrogen, ground into uniform powder using a cryogenic grinder, aliquoted into sterile centrifuge tubes, and stored at −80 °C in an ultra-low temperature freezer until further analysis. An accurately weighed portion of sample powder was digested with a mixed solution of nitric acid (HNO_3_) and perchloric acid (HClO_4_). The digest was adjusted to a constant volume with 1% nitric acid, filtered, and subsequently analyzed for Cd content using a flame atomic absorption spectrophotometer [44].

All of the reagents mentioned above were purchased from Changchun Anmei Biotechnology Co. (Changchun, China).

### 4.4. RNA Sequencing Experimental Method

Leaf samples from four treatment groups (CK, Cd, SA, and SACd), with three biological replicates per group, were collected for transcriptomic analysis. Post harvesting, leaf specimens were rinsed thoroughly with deionized water, surface-dried with absorbent paper, snap-frozen instantly in liquid nitrogen, and stored at −80 °C until RNA extraction. Total RNA was isolated from the frozen leaf samples using TRIzol reagent purchased from Invitrogen. RNA concentration, purity and structural integrity were rigorously checked using a NanoDrop spectrophotometer (Thermo Fisher Scientific, Thermo Fisher Scientific, Waltham, MA, USA) coupled with an Agilent 2100 Bioanalyzer (Agilent Technologies) to ensure qualified sequencing templates. Sequencing libraries were constructed following the manufacturer’s instructions of the VAHTS Universal V6 RNA-seq Library Prep Kit (Illumina), and paired-end sequencing was executed on an Illumina NovaSeq 6000 platform. The entire sequencing process and subsequent bioinformatics analysis were entrusted to Novogene Co., Ltd. (Beijing, China).

Raw sequencing reads were subjected to quality trimming via Trimmomatic v0.39 software to discard low-quality reads with base quality Q < 20 and reads containing poly-N fragments. The high-quality clean reads obtained were de novo assembled into transcript isoforms with Trinity v2.8.5 software; for each gene cluster sharing over 95% sequence homology, the longest transcript isoform was extracted as the representative unigene. Functional annotation of unigenes was implemented by aligning sequences against the NCBI NR, Swiss-Prot, eggNOG and KOG databases via Diamond, with an E-value threshold set at 1 × 10^−5^. GO terms and KEGG pathway annotations were further assigned based on the matched database results.

Gene expression abundance was calculated and normalized to transcript per million mapped reads (FPKM) values utilizing Bowtie and eXpress tools. Differential expression analysis across groups was conducted with the DESeq2 package, and genes meeting the criteria of |log_2_FC| ≥ 1 and adjusted *p*-value < 0.05 were screened as DEGs. Hierarchical clustering analysis of DEGs was implemented in R software (v3.2.0) to visualize the expression profiles across different sample groups. The significantly enriched GO terms and KEGG pathways among DEGs were identified via hypergeometric tests with a q-value cutoff of 0.05, followed by targeted visualization analysis.

### 4.5. qRT-PCR Validation

Quantitative real-time PCR (qPCR) was performed to validate the genes selected from the de novo transcriptome sequencing results. Reference genes were selected from the unigene set of the transcriptome data, and their expression stability was evaluated using geNorm (version 3.5) and NormFinder (version 0.953) software to ensure reliable normalization. The relative expression levels were calculated using the 2-ΔΔCT method [71]. Each qRT-PCR reaction was performed with three independent biological replicates. The qPCR validation experiment was commissioned by Beijing Biomarker Technologies Co., Ltd. (Beijing, China).

### 4.6. Data Analysis

Data were compiled and calculated using Microsoft Excel 2025. Two-way analysis of variance (ANOVA) was performed to examine the effects of four treatments on growth and related physiological indices. The least significant difference (LSD) test was used to compare significant differences between groups at *p* < 0.05, and Pearson’s correlation analysis was conducted. All figures and tables were created using Origin 2024 and GraphPad Prism 10.1.2 software. Data are expressed as mean ± standard error (mean ± SE).

## 5. Conclusions

This study reveals that SA enhances Cd tolerance in *Cornus alba* L. seedlings by strengthening root Cd sequestration, attenuating oxidative stress, and reducing Cd translocation and accumulation in stems, thus mitigating Cd toxicity. Exogenous SA application significantly enhanced osmotic adjustment and restructured the antioxidant enzyme system, effectively alleviating membrane lipid peroxidation damage. Concurrently, SA significantly increased the root capacity for Cd^2+^ immobilization, inhibited its translocation to shoots, and reduced Cd accumulation in leaves and reproductive organs, thereby mitigating the inhibition of photosynthetic function and reproductive growth. At the molecular level, transcriptome analysis revealed that SA effectively activated sucrose and starch metabolism pathways, promoting the synthesis of functional soluble sugars to provide energy and signaling support for Cd immobilization in roots. Furthermore, SA significantly activated the terpenoid biosynthesis pathway, upregulating key genes such as *HMGR* and *GGPS*. In combination with the CAT-mediated enzymatic antioxidant system, this response synergistically enhanced cellular resistance to oxidative damage under Cd stress. These findings not only advance our understanding of signaling regulatory networks in plant heavy metal stress resistance but also provide critical insights into vegetation restoration and ecological reconstruction in areas with mild to moderate Cd contamination. Future research should focus on optimizing SA application protocols for woody plants to enhance their survival and growth in Cd-contaminated soils, thereby providing a practical approach for ecological remediation projects.

## Figures and Tables

**Table 1:** Cd accumulation and transport in *Cornus alba* L. under Cd and SA application.

Treatments	Cd^2+^ Concentration (μg·g^−1^)			BCF	TF
	Shoots	Roots	Soil		
CK	0.0223 ± 0.0025 c	0.2519 ± 0.0388 c	0.3481 ± 0.0264 c	0.7878 ± 0.0136 c	0.0885 ± 0.0026 b
SA	0.0359 ± 0.0045 b	0.3483 ± 0.0158 c	5.1562 ± 0.1647 b	0.0746 ± 0.0065 d	0.1051 ± 0.0041 a
Cd	0.0602 ± 0.0010 a	28.6445 ± 1.4986 b	14.5001 ± 0.3814 a	1.9796 ± 0.0676 b	0.0021 ± 0.0001 c
SACd	0.0433 ± 0.0034 b	57.0425 ± 2.9175 a	15.1446 ± 0.4965 a	3.7694 ± 0.0593 a	0.0008 ± 0.0005 c

Note: Different lower case letters indicate significant differences between treatments (*p* < 0.05).

## Data Availability

The raw data supporting the conclusions of this article will be made available by the authors upon request.

## Supplementary Materials

The following supporting information can be downloaded at: https://www.mdpi.com/article/10.3390/plants15071081/s1, Table S1: Statistics of RNA-Seq Data in *Cornus alba* L.; Table S2: The two-way ANOVA of Growth Indices, Chlorophyll Content and Photosynthetic Parameters of *Cornus alba* L. in Response to Cadmium Stress; Table S3: The two-way ANOVA of Physiological Indices of *Cornus alba* L. in Response to Cadmium Stress; Table S4: The two-way ANOVA of Cadmium accumulation and transportof *Cornus alba* L. in Response to Cadmium Stress; Figure S1: FPKM trends in DEGs analyzed by RNA-seq and relative expression trends in DEGs verified by RT-qPCR.

## References

[1] Khan A., Jie Z., Xiangjun K., Ullah N., Short A.W., Diao Y., Zhou R., Xiong Y.-C. (2023). Pre treatment of melatonin rescues cotton seedlings from cadmium toxicity by regulating key physio-biochemical and molecular pathways. J. Hazard. Mater..

[2] Li W., Li J., Hussain K., Peng K., Yu J., Xu M., Yang S. (2024). Transporters and phytohormones analysis reveals differential regulation of ryegrass (*Lolium perenne* L.) in response to cadmium and arsenic stresses. J. Hazard. Mater..

[3] Liu M., Wang Z., Yin S. (2026). OvHMA3 enhances cadmium tolerance in *Onobrychis viciifolia* by promoting root compartmentalization via active transport. J. Hazard. Mater..

[4] Zheng Y., Zhang R., Zhu Y., Ao Q., Liu H., Li A., Lin L., Wang L. (2022). Salicylic acid improves *Nasturtium officinale* phytoremediation capability for cadmium-contaminated paddy soils. Front. Plant Sci..

[5] Tan C., Nie W., Liu Y., Wang Y., Yuan Y., Liu J., Chang E., Xiao W., Jia Z. (2024). Physiological response and molecular mechanism of *Quercus variabilis* under cadmium stress. Plant Physiol. Biochem..

[6] Küpper H., Parameswaran A., Leitenmaier B., Trtílek M., Šetlík I. (2007). Cadmium-induced inhibition of photosynthesis and long-term acclimation to cadmium stress in the hyperaccumulator *Thlaspi caerulescens*. New Phytol..

[7] Wang X., Wang W., Yang L., Jin L., Song Y., Jiang S., Qin L.J.A.E.S. (2015). Transport pathways of cadmium (Cd) and its regulatory mechanisms in plant. Acta Ecol. Sin..

[8] Zhang C., Li T., Wang Y., Lai Y., Pu T., Duan C. (2025). Mitigating cadmium toxicity in crops: Agronomic measures to regulate uptake and tolerance mechanisms. Plant Stress.

[9] Shi Y.F., Min W.F., Bai X.R., She Y.M., Tian H.T., Luo C.K. (2023). Effects of exogenous regulatory substances on physiological characteristics and gene expression of rice seedlings under alkali stress. J. Plant Nutr. Fertil..

[10] Li Y., Dong Q., Wu D., Yin Y., Du W., Guo H. (2023). A 24-epibrassinolide treatment and intercropping willow with alfalfa increase the efficiency of the phytoremediation of cadmium-contaminated soil. Sci. Total Environ..

[11] Nazir M.M., Noman M., Ahmed T., Ali S., Ulhassan Z., Zeng F., Zhang G. (2022). Exogenous calcium oxide nanoparticles alleviate cadmium toxicity by reducing Cd uptake and enhancing antioxidative capacity in barley seedlings. J. Hazard. Mater..

[12] Tu D., Tian X., Liang C., Zhou P., Wang Y., Xing D., Liu Y. (2025). Physiological and biochemical responses of pepper (*Capsicum annuum*) to cadmium stress: The mitigating effects of exogenous abscisic acid. Ecotoxicol. Environ. Saf..

[13] Rhimi N., Hajji M., Elkhouni A., Ksiaa M., Rabhi M., Lefi E., Smaoui A., Hessini K., Hamzaoui A.H., Cabassa-Hourton C. (2024). Silicon Reduces Cadmium Accumulation and Improves Growth and Stomatal Traits in Sea Barley (*Hordeum marinum* Huds.) Exposed to Cadmium Stress. J. Soil Sci. Plant Nutr..

[14] Li C., Liu J., Wei Z., Cheng Y., Shen Z., Xin Z., Huang Y., Wang H., Li Y., Mu Z. (2023). Exogenous melatonin enhances the tolerance of tiger nut (*Cyperus esculentus* L.) via DNA damage repair pathway under heavy metal stress (Cd2+) at the sprout stage. Ecotoxicol. Environ. Saf..

[15] Jian L., Jingchun L., Chongling Y., Daolin D., Haoliang L. (2019). The alleviation effect of iron on cadmium phytotoxicity in mangrove A. marina. Alleviation effect of iron on cadmium phytotoxicity in mangrove *Avicennia marina* (Forsk.) Vierh. Chemosphere.

[16] Han Y., Chen G., Chen Y., Shen Z. (2015). Cadmium Toxicity and Alleviating Effects of Exogenous Salicylic Acid in *Iris hexagona*. Bull. Environ. Contam. Toxicol..

[17] Peng Y., Yang J., Li X., Zhang Y. (2021). Salicylic Acid: Biosynthesis and Signaling. Annu. Rev. Plant Biol..

[18] Shi A., Xu J., Shao Y., Alwathnani H., Rensing C., Zhang J., Xing S., Ni W., Zhang L., Yang W. (2024). Salicylic Acid’s impact on *Sedum alfredii* growth and cadmium tolerance: Comparative physiological, transcriptomic, and metabolomic study. Environ. Res..

[19] Singh A.P., Dixit G., Mishra S., Dwivedi S., Tiwari M., Mallick S., Pandey V., Trivedi P.K., Chakrabarty D., Tripathi R.D. (2015). Salicylic acid modulates arsenic toxicity by reducing its root to shoot translocation in rice (*Oryza sativa* L.). Front. Plant Sci..

[20] Hayat U., ul din K., Ahmad M., Zulfiqar U., Sajjad M., Maqsood M.F., Soufan W., Prasad P.V.V., Djalovic I. (2025). Salicylic acid confers cadmium tolerance in wheat by regulating photosynthesis, yield and ionic homeostasis. Sci. Rep..

[21] Li Q., Guan C., Zhao Y., Duan X., Yang Z., Zhu J. (2023). Salicylic acid alleviates Zn-induced inhibition of growth via enhancing antioxidant system and glutathione metabolism in alfalfa. Ecotoxicol. Environ. Saf..

[22] Faraz A., Faizan M., Sami F., Siddiqui H., Hayat S. (2020). Supplementation of Salicylic Acid and Citric Acid for Alleviation of Cadmium Toxicity to *Brassica juncea*. J. Plant Growth Regul..

[23] Dadhich A., Sharma M.M. (2025). Remediation of cadmium-contaminated soil using *Bacopa monnieri* (L.) Wettst. Synergistic role of salicylic and jasmonic acids in Phytostabilisation and neuroprotective bacoside A biosynthesis. Curr. Res. Biotechnol..

[24] Gupta S., Seth C.S. (2021). Salicylic acid alleviates chromium (VI) toxicity by restricting its uptake, improving photosynthesis and augmenting antioxidant defense in *Solanum lycopersicum* L.. Physiol. Mol. Biol. Plants.

[25] Tang W., Liang L., Yang H., Yu X., Ye X., Xie Y., Li R., Lin L., Huang Z., Sun B. (2024). Exogenous salicylic acid reduces cadmium content in spinach (*Spinacia oleracea* L.) shoots under cadmium stress. BMC Plant Biol..

[26] Luo S., Wang K., Li Z., Li H., Shao J., Zhu X. (2022). Salicylic Acid Enhances Cadmium Tolerance and Reduces Its Shoot Accumulation in *Fagopyrum tataricum* Seedlings by Promoting Root Cadmium Retention and Mitigating Oxidative Stress. Int. J. Mol. Sci..

[27] Xu X.H., Huang D.Z., Wang L.F. (2009). Effects of Pb, Cd stress in soil on the growth and physiological and biochemical characteristics of *Swida alba*. J. Soil Water Conserv..

[28] Li J., Tian Q. (2022). Leaf morphology and photosynthetic physiological characteristics of six garden plants in Lanzhou. J. Northwest A&F Univ.-Nat. Sci. Ed..

[29] Yu J., Chen H.W., Yan H.W. (2015). Study on carbon sink functions of garden plants in urban areas of Shenyang, Liaoning. J. Cent. South Univ. For. Technol..

[30] Wu Y., Mu L. (2008). Effect of soil Pb, Cd stress on the growth, physiological and accumulation characteristics of four ornamental trees. J. Trop. Crops.

[31] Chai C., Mu L., Liang M., Wang R. (2012). Physiological responses of six northern greening shrubs to water stress. J. Northeast. For. Univ..

[32] Chai C., Ma L., Mu L. (2010). Responses of moisture parameters of six landscape shrub species in northern China to drought stress. J. Northeast. For. Univ..

[33] Tang W., Liang L., Xie Y., Li X., Lin L., Huang Z., Sun B., Sun G., Tu L., Li H. (2023). Foliar application of salicylic acid inhibits the cadmium uptake and accumulation in lettuce (*Lactuca sativa* L.). Front. Plant Sci..

[34] Popova L.P., Maslenkova L.T., Yordanova R.Y., Ivanova A.P., Krantev A.P., Szalai G., Janda T. (2009). Exogenous treatment with salicylic acid attenuates cadmium toxicity in pea seedlings. Plant Physiol. Biochem..

[35] Huang Z., Song X., Song J., Su L., Meng S., Yu X., Liang K., Huang H., Zhang F., Li H. (2025). Physiological and transcriptomic analysis of purple flowering stalks (*Brassica campestris* var. *purpurea*) under cadmium stress and exogenous glutathione application. Plant Physiol. Biochem..

[36] Peng G., Dexiang T., Hongling H., Gang C., Chuang L., Donghao L., Wen D.E.N.G. (2025). Response of growth and nutrient absorption of *Phoebe zhennan* saplings to cadmium stress and its bioaccumulation characteristics for cadmium. J. Northwest A&F Univ.-Nat. Sci. Ed..

[37] Shaari N.E.M., Tajudin M.T.F.M., Khandaker M.M., Majrashi A., Alenazi M.M., Abdullahi U.A., Mohd K.S. (2024). Cadmium toxicity symptoms and uptake mechanism in plants: A review. Braz. J. Biol..

[38] Zhou J., Han P.-P., Pan Y.-Z., Wu M.-X., Zhao Y., Jia Y., Jiang B.-B., Zhang L., Xu Q., Liu S.-L. (2021). Effects of cadmium stress on photosynthetic physiology and chlorophyll fluorescence in *Solanum nigrum* and *Solanum americanum*. J. Agro-Environ. Sci..

[39] Belkhadi A., Hediji H., Abbes Z., Nouairi I., Barhoumi Z., Zarrouk M., Chaïbi W., Djebali W. (2010). Effects of exogenous salicylic acid pre-treatment on cadmium toxicity and leaf lipid content in *Linum usitatissimum* L.. Ecotoxicol. Environ. Saf..

[40] Li L., Liu T., Deng Q., Lin L., Wang X., Peng J., Mao M. (2019). Effects of exogenous salicylic acid on growth and cadmium accumulation of *Ziziphus acidojujuba* seedlings under cadmium pollution. J. Sichuan Agric. Univ..

[41] Muradoglu F., Gundogdu M., Ercisli S., Encu T., Balta F., Jaafar H.Z.E., Zia-Ul-Haq M. (2015). Cadmium toxicity affects chlorophyll a and b content, antioxidant enzyme activities and mineral nutrient accumulation in strawberry. Biol. Res..

[42] Ogunkunle C.O., Balogun G.Y., Olatunji O.A., Han Z., Adeleye A.S., Awe A.A., Fatoba P.O. (2023). Foliar application of nanoceria attenuated cadmium stress in okra (*Abelmoschus esculentus* L.). J. Hazard. Mater..

[43] Zhang X., Yang M., Yang H., Pian R., Wang J., Wu A.-M. (2024). The Uptake, Transfer, and Detoxification of Cadmium in Plants and Its Exogenous Effects. Cells.

[44] Xu Y.-f., Chen D.-w., Ma J., Gao R.-c., Bai J., Hou Q.-z. (2024). Transcriptomic and physiological analyses of *Symphytum officinale* L. in response to multiple heavy metal stress. Ecotoxicol. Environ. Saf..

[45] Dai F., Luo G., Li Z., Wei X., Wang Z., Lin S., Tang C. (2020). Physiological and transcriptomic analyses of mulberry (*Morus atropurpurea*) response to cadmium stress. Ecotoxicol. Environ. Saf..

[46] Lux A., Martinka M., Vaculík M., White P.J. (2011). Root responses to cadmium in the rhizosphere: A review. J. Exp. Bot..

[47] Verbruggen N., Hermans C., Schat H. (2009). Molecular mechanisms of metal hyperaccumulation in plants. New Phytol..

[48] Baker A.J.M. (1981). Accumulators and excluders—Strategies in the response of plants to heavy metals. J. Plant Nutr..

[49] Chen Z.j., Huang J., Li S., Shao J.F., Shen R.F., Zhu X.F. (2023). Salylic acid minimize cadmium accumulation in rice through regulating the fixation capacity of the cell wall to cadmium. Plant Sci..

[50] Zheng Z., Ghouri F., Ali S., Xia W., Li Z., Sun L., Shahid M.Q. (2025). Sucrose transporters and calcium oxide mitigate cadmium toxicity in rice by regulating carbohydrate metabolism, metal transporters, and oxidative stress. Environ. Chem. Ecotoxicol..

[51] Sharma S.S., Dietz K.J. (2006). The significance of amino acids and amino acid-derived molecules in plant responses and adaptation to heavy metal stress. J. Exp. Bot..

[52] Sharma A., Sidhu G.P., Araniti F., Bali A.S., Shahzad B., Tripathi D.K., Brestic M., Skalicky M., Landi M. (2020). The Role of Salicylic Acid in Plants Exposed to Heavy Metals. Molecules.

[53] Marefat E., Shabani L., Amooaghaie R. (2012). Interaction effect of cadmium and salicylic acid on proline and antioxidant enzyme activity in Soybean. Malays. Appl. Biol..

[54] El-Tayeb M.A., El-Enany A.E., Ahmed N.L. (2006). Salicylic acid-induced adaptive response to copper stress in sunflower (*Helianthus annuus* L.). Plant Growth Regul..

[55] Elazab D.S., El-Mahdy M., Youssef M., Eissa M.A., Amro A., Lambardi M. (2023). Assessment of Salicylic Acid as a Pretreatment on Alleviating Cadmium Toxicity on In Vitro Banana Shoots. J. Plant Growth Regul..

[56] Eh T.-J., Jiang Y., Jiang M., Li J., Lei P., Ji X., Kim H.-I., Zhao X., Meng F. (2025). The role of trehalose metabolism in plant stress tolerance. J. Adv. Res..

[57] Bolouri Moghaddam M.R., Van den Ende W. (2012). Sugars and plant innate immunity. J. Exp. Bot..

[58] Niu Z., Liu L., Pu Y., Ma L., Wu J., Hu F., Fang Y., Li X., Sun W., Wang W. (2021). iTRAQ-based quantitative proteome analysis insights into cold stress of Winter Rapeseed (*Brassica rapa* L.) grown in the field. Sci. Rep..

[59] Shang Q., Wei Z., Fang L., Huang D., Pan X. (2025). Integrated physiological, biochemical, transcriptomic, and metabolomic analysis reveals the mechanism of silicon in alleviating cadmium stress in *Juglans sigillata*. Ind. Crops Prod..

[60] Torun H., Cetin B., Stojnic S., Petrík P. (2024). Salicylic acid alleviates the effects of cadmium and drought stress by regulating water status, ions, and antioxidant defense in *Pterocarya fraxinifolia*. Front. Plant Sci..

[61] Hediji H., Kharbech O., Massoud M.B., Boukari N., Debez A., Chaibi W., Chaoui A., Djebali W. (2021). Salicylic acid mitigates cadmium toxicity in bean (*Phaseolus vulgaris* L.) seedlings by modulating cellular redox status. Environ. Exp. Bot..

[62] Lin Y.-P., Charng Y.-y. (2021). Chlorophyll dephytylation in chlorophyll metabolism: A simple reaction catalyzed by various enzymes. Plant Sci..

[63] Romer J., Gutbrod K., Schuppener A., Melzer M., Müller-Schüssele S.J., Meyer A.J., Dörmann P. (2024). Tocopherol and phylloquinone biosynthesis in chloroplasts requires the phytol kinase VITAMIN E PATHWAY GENE5 (VTE5) and the farnesol kinase (FOLK). Plant Cell.

[64] Wang Y., Zhang S., Ma Y., Du X., Zong Q., Lin D., Lai M., Huang T., Luo Q., Yang L. (2024). Solvent effects on terpenoid compositions and antioxidant activities of *Cinnamomum camphora* (L.) J. Presl extracts and the main antioxidant agent evaluation through in vitro and in vivo assay. Chem. Biol. Technol. Agric..

[65] Xu Y.W. (2014). Effects of salicylic acid on monoterpene production and antioxidant systems in *Houttuynia cordata*. Afr. J. Biotechnol..

[66] Guo P., Wang Z., Xia Y., Wang X. (2025). Effect of Salicylic Acid on Triterpenoid Biosynthesis and Reactive Oxygen Species Content in *Ganoderma applanatum* Mycelium. Food Sci..

[67] Jiang L., Yun M., Ma Y., Qu T. (2024). Melatonin Mitigates Water Deficit Stress in *Cenchrus alopecuroides* (L.) Thunb through Up-Regulating Gene Expression Related to the Photosynthetic Rate, Flavonoid Synthesis, and the Assimilatory Sulfate Reduction Pathway. Plants.

[68] Zhou X., Jin T., Li T., An Y., Dai X., Zhao C., Qu T. (2025). *Euphorbia marginata* Alleviate Heavy Metal Ni-Cu Combined Stress by Regulating the Synthesis of Signaling Factors and Flavonoid Organisms. Plants.

[69] Mittra P.K., Rahman M.A., Roy S.K., Kwon S.-J., Yun S.H., Kun C., Zhou M., Katsube-Tanaka T., Shiraiwa T., Woo S.-H. (2024). Deciphering proteomic mechanisms explaining the role of glutathione as an aid in improving plant fitness and tolerance against cadmium-toxicity in *Brassica napus* L.. J. Hazard. Mater..

[70] Kramer G.F., Norman H.A., Krizek D.T., Mirecki R.M. (1991). influence of UV-B radiation on polyamines, lipid peroxidation and membrane lipids in cucumber. Phytochemistry.

[71] Livak K.J., Schmittgen T.D. (2001). Analysis of relative gene expression data using real-time quantitative PCR and the 2(-Delta Delta C(T)) Method. Methods.

