# Supplementary material for: Salicylic Acid Enhances Cadmium Tolerance in Cornus alba L. Seedlings Through Leaf Transcriptional Regulation and Enhanced Root Heavy Metal Sequestration
Source: Plants (Basel). 2026 Apr 1;15(7):1081. doi: 10.3390/plants15071081 (PMC13074589; doi:10.3390/plants15071081)
Supplement: Supplementary file 1 [file plants-15-01081-s001.zip › Figure S1. FPKM trends in DEGs analyzed by RNA-seq and relative expression trends in DEGs verified by RT-qPCR.pdf]

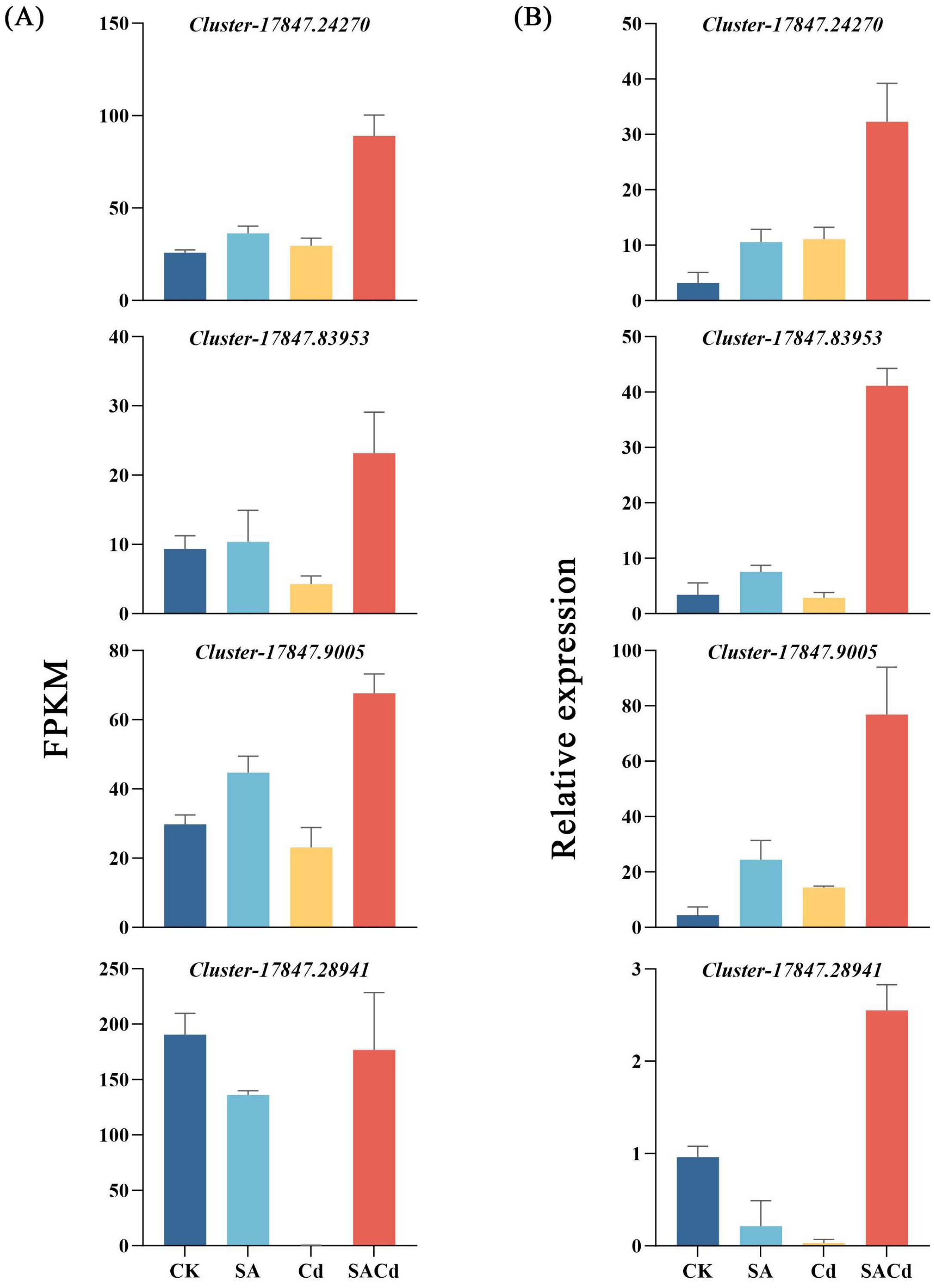

Figure S1. FPKM trends in DEGs analyzed by RNA-seq and relative expression trends in DEGs verified by RT-qPCR
